# Associations between fine-root traits and soil nutrient profile differentiation during degradation of *Populus simonii* shelterbelts in sandy areas

**DOI:** 10.3389/fpls.2026.1865203

**Published:** 2026-07-09

**Authors:** Haibing Wang, Jin Ni, Xue Chen, Hejun Zuo, Xinghua Zhao

**Affiliations:** 1 State Key Laboratory of Water Engineering Ecology and Environment in Arid Area, Inner Mongolia Agricultural University, Hohhot, China; 2 Inner Mongolia Key Laboratory of Aeolian Physics and Desertification Control Engineering, College of Desert Control Science and Engineering, Inner Mongolia Agricultural University, Hohhot, Inner Mongolia Autonomous Region, China; 3 Inner Mongolia Hanggin Desert Ecosystem Positioning Research Station, Hohhot, Inner Mongolia Autonomous Region, China

**Keywords:** belowground processes, degradation level, fine-root traits, *Populus simonii* shelterbelts, soil nutrient profile differentiation

## Abstract

Understanding belowground changes during stand degradation is essential for the restoration of degraded shelterbelts in sandy areas. This study investigated *Populus simonii* shelterbelts across four degradation levels (Healthy, Lightly degraded, Moderately degraded, and Severely degraded) in the Kubuqi Desert. We analyzed the relationships between fine-root traits and soil nutrient variation along the 0–100 cm soil profile to characterize their associations during degradation. The results showed that, as degradation intensified, fine-root activity and biomass decreased significantly, whereas lignin content and the lignin-to-cellulose ratio peaked at the Moderately degraded stage, indicating a shift in fine-root resource-use strategy from an acquisitive strategy to a more conservative one. Soil organic matter, total nitrogen, and alkali-hydrolyzable nitrogen generally declined along the soil profile, with the surface layer showing the strongest response. Total nitrogen, total phosphorus, and alkali-hydrolyzable nitrogen were the key soil factors driving fine-root trait variation. Path analysis further showed that changes in total soil nutrients may contribute to reduced fine-root activity and trait differentiation by affecting the supply of available nutrients and fine-root chemical composition. Overall, fine-root trait changes were significantly associated with soil nutrient profile differentiation, and the Moderately degraded stage exhibited a more pronounced transition in belowground functioning. This study deepens our understanding of the degradation of *Populus simonii* shelterbelts in sandy areas from a belowground perspective and provides a basis for the ecological diagnosis and restoration of degraded shelterbelts.

## 1 Introduction

Against the backdrop of global climate change, the stability of forest ecosystems in arid and semi-arid regions is facing persistent challenges (Chen et al., 2026). In particular, artificial forests in sandy areas, which serve functions such as windbreak and sand fixation, soil and water conservation, and farmland protection, exhibit pronounced spatial heterogeneity and varying degrees of degradation (Breshears et al., 2005; Allen et al., 2010). The combined effects of long-term water deficit, poor soil fertility, and wind erosion not only suppress stand growth, but also accelerate the accumulation of declining individuals and the deterioration of stand structure, thereby threatening the persistence and ecological functioning of shelterbelts (Zhu et al., 2022; Wang J, et al., 2024; Chen et al., 2025). In sandy-area plantations, degradation is reflected not only in aboveground decline, but also potentially in changes in fine-root functioning and soil resource conditions. Understanding how these two processes are linked is critical for elucidating the belowground basis of plantation degradation.

Current understanding of plantation degradation has largely focused on aboveground components, mainly involving changes in photosynthetic physiology and water-use efficiency (Broeckx et al., 2014), canopy decline, and growth reduction (Song et al., 2021), whereas the role of belowground systems in the degradation process has received relatively limited attention. Fine roots are the primary organs for water and nutrient uptake and are also the belowground components that first perceive and respond to soil stress (Freschet et al., 2021). Fine-root activity reflects root metabolic status and resource acquisition capacity (Kalra et al., 2024; Li et al., 2021), whereas lignin, cellulose, and their ratio characterize structural investment and resource-use strategies in fine roots (Lyu et al., 2023; Saha et al., 2023). As environmental stress intensifies, fine roots may maintain survival by reducing metabolic activity and increasing structural investment, but such adjustments are often accompanied by declines in resource acquisition capacity (Han et al., 2022; Hou et al., 2024).

Changes in fine-root functioning are accompanied by shifts in understory soil nutrient conditions and vertical nutrient distribution patterns. In arid and semi-arid regions, the vertical stratification of soil nutrients is particularly pronounced (Chen et al., 2021; Zhou et al., 2023). Declining surface vegetation cover and intensified wind erosion can accelerate the loss of surface organic matter and fine particles. At the same time, changes in root distribution and uptake intensity, together with solute migration and redistribution, may also alter the profile distribution and availability of nitrogen, phosphorus, and potassium (Song et al., 2024; Yang et al., 2021). Previous studies have shown that changes in soil nutrient conditions can significantly affect fine-root biomass and its vertical distribution in poplar plantations (Geng et al., 2021), indicating a close linkage between soil resource conditions and fine-root responses, both of which may jointly participate in the process of stand degradation. Although root–soil relationships in forest and plantation ecosystems have been widely studied, the relationship between fine-root trait variation and soil nutrient conditions across degradation gradients in shelterbelts remains insufficiently understood. In particular, the associations between changes in fine-root activity, biomass, and lignin–cellulose traits and soil nutrient conditions remain unclear.

*Populus simonii* is an important tree species used in artificial shelterbelts in the wind-eroded and sandy regions of northern China and has been widely planted in arid and semi-arid areas (Liang et al., 2022; Wang et al., 2025). Despite its strong environmental adaptability, its stands may still exhibit marked decline under long-term limitations of water and nutrients, as reflected by reduced canopy closure, increased dead branches, and a higher proportion of declining trees (Ji et al., 2020; Fang et al., 2021). Our previous field surveys revealed clear degradation differentiation among *Populus simonii* shelterbelts in the Kubuqi Desert. Even for shelterbelt tree species with relatively strong environmental adaptability, stand stability may still decline under persistent drought stress, while the key ecological basis underlying this process remains insufficiently understood. However, in *Populus simonii* shelterbelts in sandy areas, it remains unclear whether fine-root activity, biomass, and chemical composition change systematically with increasing degradation, and whether profile-scale differentiation in soil nutrients exhibits coordinated belowground responses with fine-root variation. This lack of understanding has, to some extent, limited our interpretation of the degradation process and its ecological basis.

Accordingly, this study focused on *Populus simonii* shelterbelts across different degradation levels in the Kubuqi Desert to analyze the associations between fine-root trait changes and soil nutrient profile variation and to explore their belowground implications for stand degradation. Specifically, this study addressed the following questions: (1) Do fine-root activity, biomass, and chemical composition in *Populus simonii* change systematically across the degradation levels? (2) How do soil nutrients along the 0–100 cm profile respond to stand degradation? (3) Are fine-root trait changes significantly associated with variation in soil nutrient conditions, and can these associations provide a belowground explanation for stand degradation? This study aims to provide a scientific basis for identifying belowground processes and guiding the ecological restoration of degraded shelterbelts in sandy areas.

## 2 Materials and methods

### 2.1 Overview of the study area

This study was conducted in a representative shelterbelt plantation area in the Kubuqi Desert, Inner Mongolia. The study area is located along the northern edge of the central Kubuqi Desert (40°18′30″–40°26′20″N, 109°46′40″–110°04′30″E) and has a temperate arid continental climate, characterized by intense wind–sand activity. The vegetation is dominated by planted shelterbelts and shrublands. Annual precipitation is approximately 250 mm but is unevenly distributed throughout the year, with most rainfall occurring between June and September (accounting for about 70–85% of the annual total). Potential evaporation far exceeds precipitation, and strong winds frequently occur in spring, accompanied by intense wind–sand activity, imposing long-term water stress and wind erosion on shelterbelt growth and stability. The study area is characterized by shifting and semi-fixed dunes and gently undulating sandy terrain. The soil is primarily aeolian sandy soil with a loose texture and poor water- and nutrient-retention capacity.

**Figure 1:**
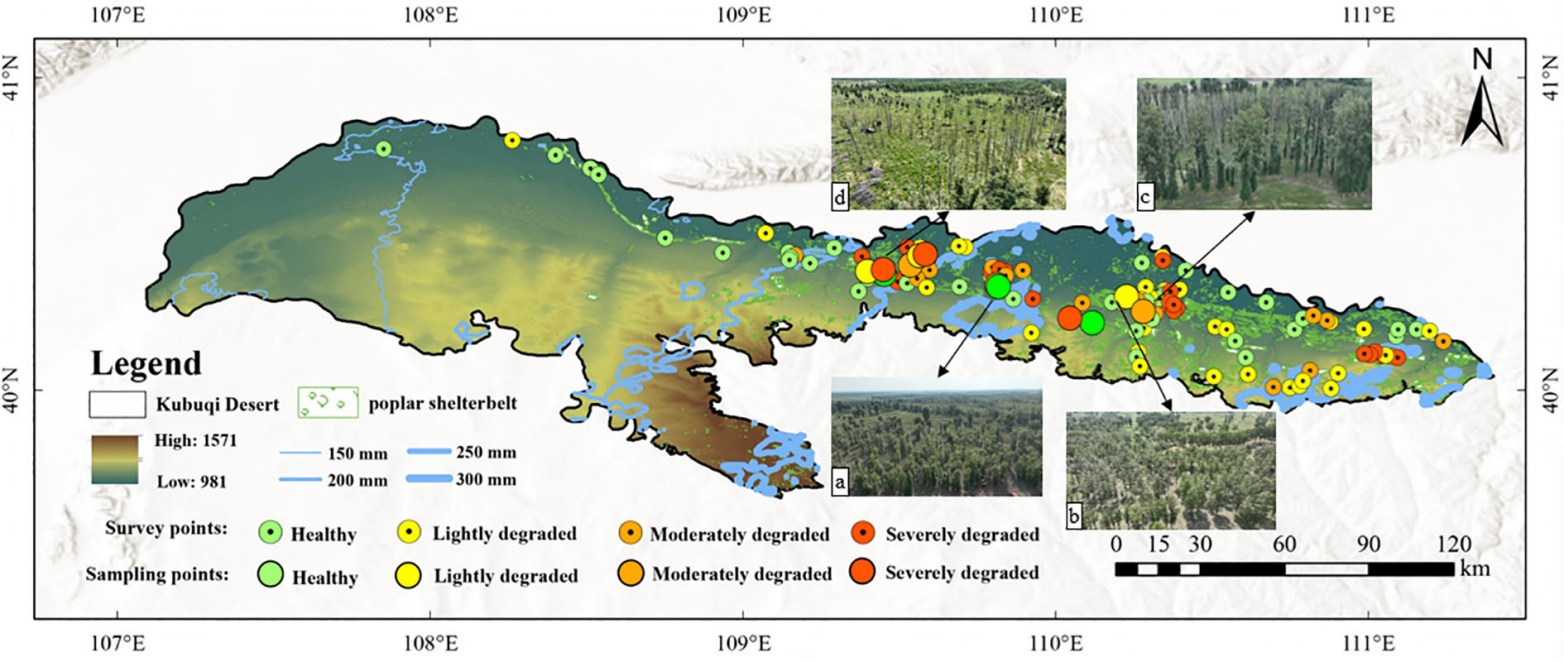
Locations of poplar shelterbelt plots under different degradation levels: **(a)** healthy; **(b)** lightly degraded; **(c)** moderately degraded; and **(d)** severely degraded.

Preliminary field surveys indicate that poplar shelterbelts throughout the Kubuqi Desert are generally experiencing varying degrees of degradation. Among the 131 surveyed plots, approximately 40% were classified as moderately or severely degraded based on the proportions of dead and dying trees and dead branches (**Figure 1**). The specific classification criteria are provided in **Table 1**. Given the marked east–west differences in precipitation across the Kubuqi Desert—with the western region receiving less precipitation (approximately 150 mm annually) and the eastern region receiving more (approximately 300 mm annually)—we selected 12 typical *Populus simonii* shelterbelts in the central Kubuqi Desert as study sites to minimize the influence of climatic variation on the results. All poplar shelterbelts in the study area were established more than 30 years ago and have been managed under relatively consistent practices. Due to the combined effects of site heterogeneity and external disturbances, the shelterbelts have undergone varying degrees of degradation. In less degraded plots, stand structure remains relatively intact, the canopy is more continuous, and understory cover is denser; as degradation intensifies, stand density decreases, and the proportion of dead and declining trees increases. Common understory species include *Artemisia ordosica*, *Calligonum mongolicum*, and *Caragana korshinskii*.

**Table 1:** Characteristics of the sample plots.

Degradation level	Degree of decline in protective functions	Percentage of dead and dying trees	Proportion of dead branches	Mean canopy closure
Healthy	No decline	< 5%	< 25%	0.47 ± 0.05
Lightly degraded	Slight decline	5–10%	25–50%	0.42 ± 0.04
Moderately degraded	Significant decline	11–40%	50–75%	0.34 ± 0.04
Severely degraded	Severe decline	> 40%	75–100%	0.21 ± 0.07

### 2.2 Plot delineation and sample collection

Field sampling was conducted in August 2025, during the growing season of *Populus simonii*. To reduce the short-term influence of rainfall on soil nutrient contents, sampling was not conducted shortly after rainfall events, and efforts were made to maintain relatively comparable moisture conditions among plots.

At each site, one standard 20 m × 20 m plot was established to record the number of dead and dying trees in the dominant canopy layer, as well as the proportion of dead branches. In this study, “dead trees” were defined as trees that had died completely, whereas “dying trees” were defined as trees with more than two-thirds crown dieback or trees that were completely withered and near death. Based on the proportion of dead and dying trees relative to the total number of trees per unit area, together with the proportion of dead branches, the plots were classified into four degradation levels: Healthy, Lightly degraded, Moderately degraded, and Severely degraded. Three replicate plots were established for each degradation level, resulting in a total of 12 plots, and three sample trees were selected in each plot for excavation. The specific classification criteria are shown in **Table 1** (Chen et al., 2026; Dai et al., 2024).

For each sample tree, a 1 m × 1 m × 1 m soil profile was excavated 10 cm south of the trunk. Soil samples were collected from the 0–100 cm profile at 20 cm intervals, namely 0–20, 20–40, 40–60, 60–80, and 80–100 cm. At each depth, soil from the four cardinal directions (north, south, east, and west) around the sample tree was combined into one composite sample. Therefore, a total of 180 soil samples were obtained, corresponding to 4 degradation levels × 3 replicate plots per degradation level × 3 sample trees per plot × 5 soil depths. Soil samples were used to determine nutrient indicators, including organic matter, total nitrogen, total phosphorus, total potassium, alkali-hydrolyzable nitrogen, available phosphorus, and available potassium.

During excavation, the excavated soil blocks were sieved to collect 180 root samples for biomass determination. After collection, roots were gently separated from the soil, washed with deionized water, stored in a vehicle-mounted refrigerator at 4 °C, and transported to the laboratory for sorting. Following common criteria used in fine-root studies, roots with diameters <2 mm were defined as fine roots (Puri et al., 1994). Live fine roots were identified based on their lighter color, elasticity, intact cortex, and moist appearance, whereas dead roots were identified based on their darker color, brittle texture, loose or detached cortex, and loss of elasticity. These criteria are commonly used in root sorting procedures (Bauhus and Messier, 1999; Meinen et al., 2009). In the laboratory, only live fine roots with diameters <2 mm were selected for measurements of fine-root activity and chemical traits.

### 2.3 Sample analysis

After the soil and root samples were transported to the laboratory, the relevant parameters were determined separately. Specifically, soil organic matter (SOM) was determined using the potassium dichromate oxidation–external heating method; total nitrogen (TN) was determined using the Kjeldahl method; total phosphorus (TP) was determined using the acid-soluble molybdenum–antimony colorimetric method; total potassium (TK) was determined using the acid-soluble flame photometric method; alkali-hydrolyzable nitrogen (AN) was determined using the alkali diffusion method; available phosphorus (AP) was determined using the 0.5 mol·L^−1^ NaHCO_3_ extraction–molybdenum–antimony colorimetric method; and available potassium (AK) was determined using the 1.0 mol·L^−1^ NH_4_OAc extraction–flame photometric method. For root traits, fine-root activity was determined using the TTC reduction method; lignin content was measured using a micro-assay kit; cellulose content was measured using an anthrone colorimetric assay kit; and biomass was determined using the oven-drying method.

### 2.4 Statistical analysis methods

**Figure 2:**
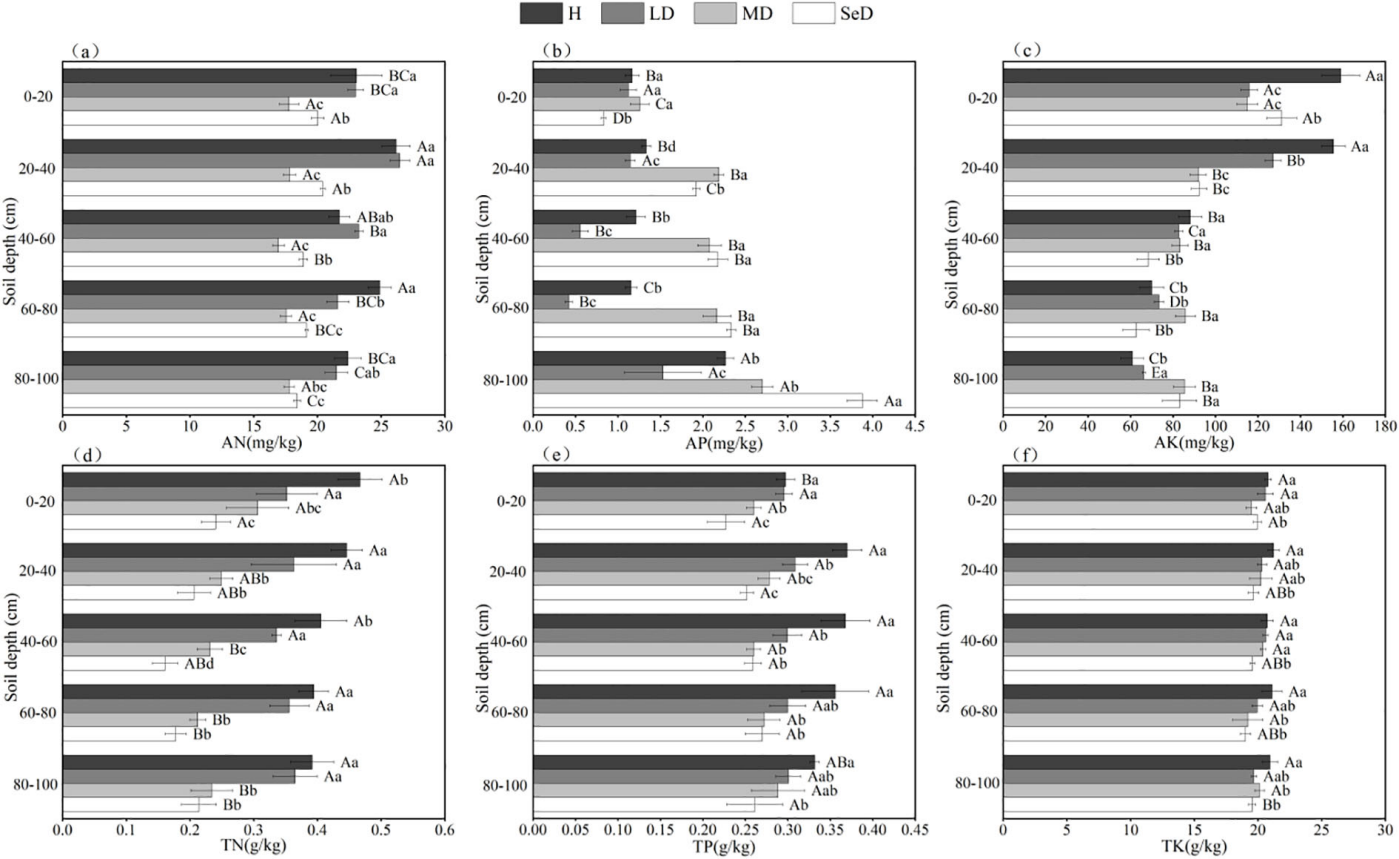
Soil nutrient characteristics across soil depths under different degradation levels. Different lowercase letters indicate significant differences among degradation levels within the same soil layer (P < 0.05), whereas the same lowercase letters indicate no significant differences. Different uppercase letters indicate significant differences among soil layers within the same degradation level (P < 0.05), whereas the same uppercase letters indicate no significant differences (P > 0.05). H, LD, MD, and SeD represent healthy, lightly degraded, moderately degraded, and severely degraded plots, respectively. **(a)** AN; **(b)** AP; **(c)** AK; **(d)** TN; **(e)** TP; **(f)** TK.

A one-way ANOVA was used to examine differences in fine-root activity, biomass, lignin, cellulose, and the L/C ratio among degradation levels. Within each soil layer, differences among degradation levels were tested by one-way ANOVA, and within each degradation level, differences among soil depths were tested by one-way ANOVA. Tukey’s multiple comparison test was performed at P < 0.05. Statistical analyses were performed using IBM SPSS Statistics 27. Principal component analysis (PCA) was conducted in R (v4.5.2) to examine the overall differentiation patterns of soil nutrient and fine-root trait indicators across soil depths under different degradation levels. Based on the soil nutrient profile results (**Figure 2**) and the supporting results in **Supplementary Table 1**; nutrient characteristics differed most markedly in the 0–20 cm soil layer, whereas the 20–100 cm layers showed relatively similar variation patterns. Therefore, the 0–20 cm indicators were retained separately, and the mean values of the 20–100 cm layers were used as composite indicators of deeper soil conditions. Redundancy analysis (RDA) was used to examine the relationships between soil nutrient factors and fine-root traits. The envfit function was applied to assess the strength and significance of the associations between environmental factors, response variables, and the ordination pattern using 999 permutations. A PLS-SEM path model of “degradation level–soil nutrients–root traits” (GOF = 0.73) was constructed to analyze the direct and indirect effects of degradation on soil nutrients and fine-root responses.

## 3 Results

### 3.1 Characteristics of fine-root traits of *Populus simonii* under different degradation levels

**Figure 3:**
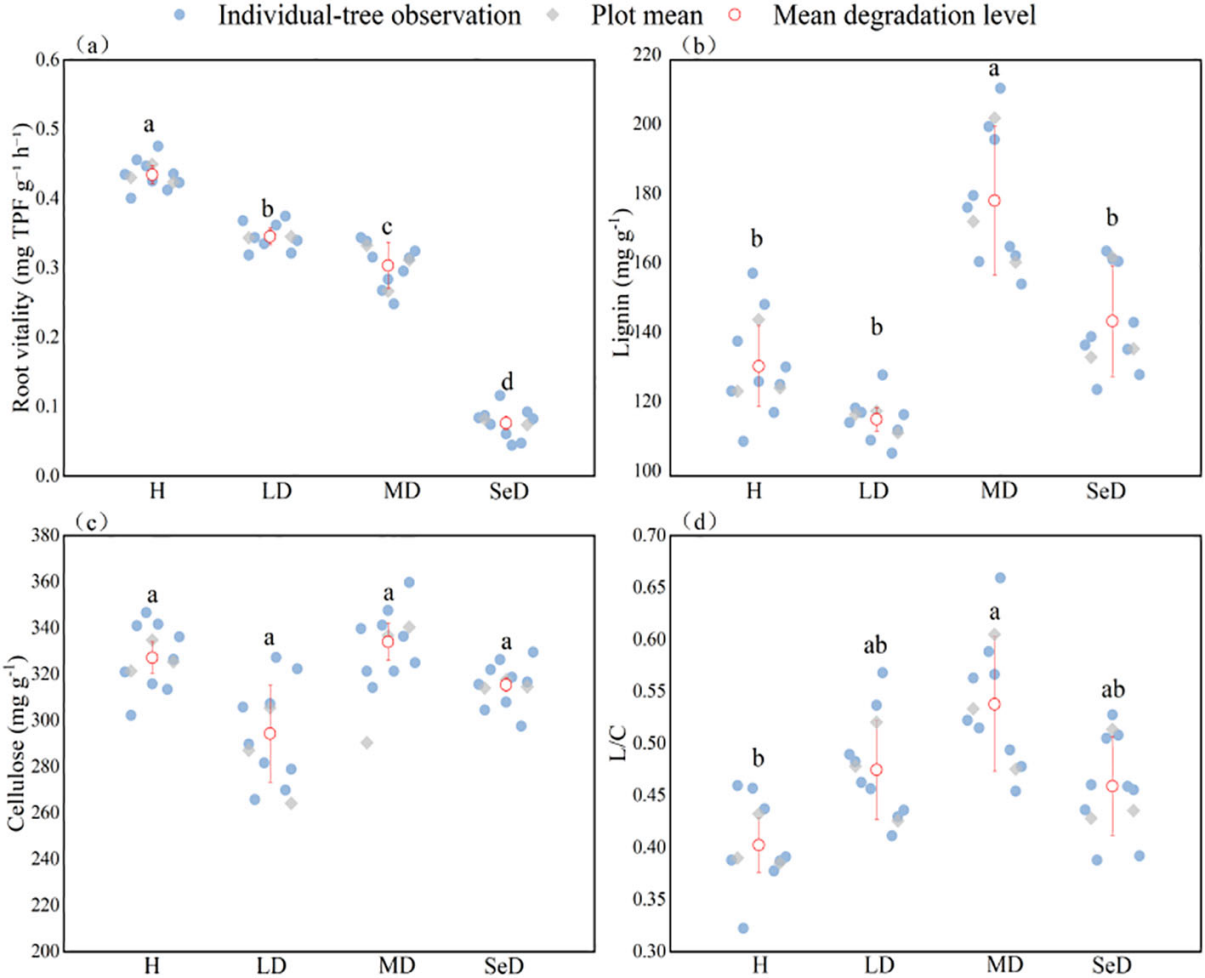
Characteristics of fine-root traits under different degradation levels. Different lowercase letters indicate significant differences among degradation levels (P < 0.05), whereas the same lowercase letters indicate no significant differences (P > 0.05). H, LD, MD, and SeD represent healthy, lightly degraded, moderately degraded, and severely degraded plots, respectively.

As degradation intensified, fine-root activity in *Populus simonii* decreased significantly, with significant differences among degradation levels (P < 0.05; **Figure 3**). Correspondingly, fine-root chemical composition also changed markedly: lignin content and the L/C ratio peaked at the Moderately degraded stage, whereas cellulose content was lowest at the Lightly degraded stage.

**Figure 4:**
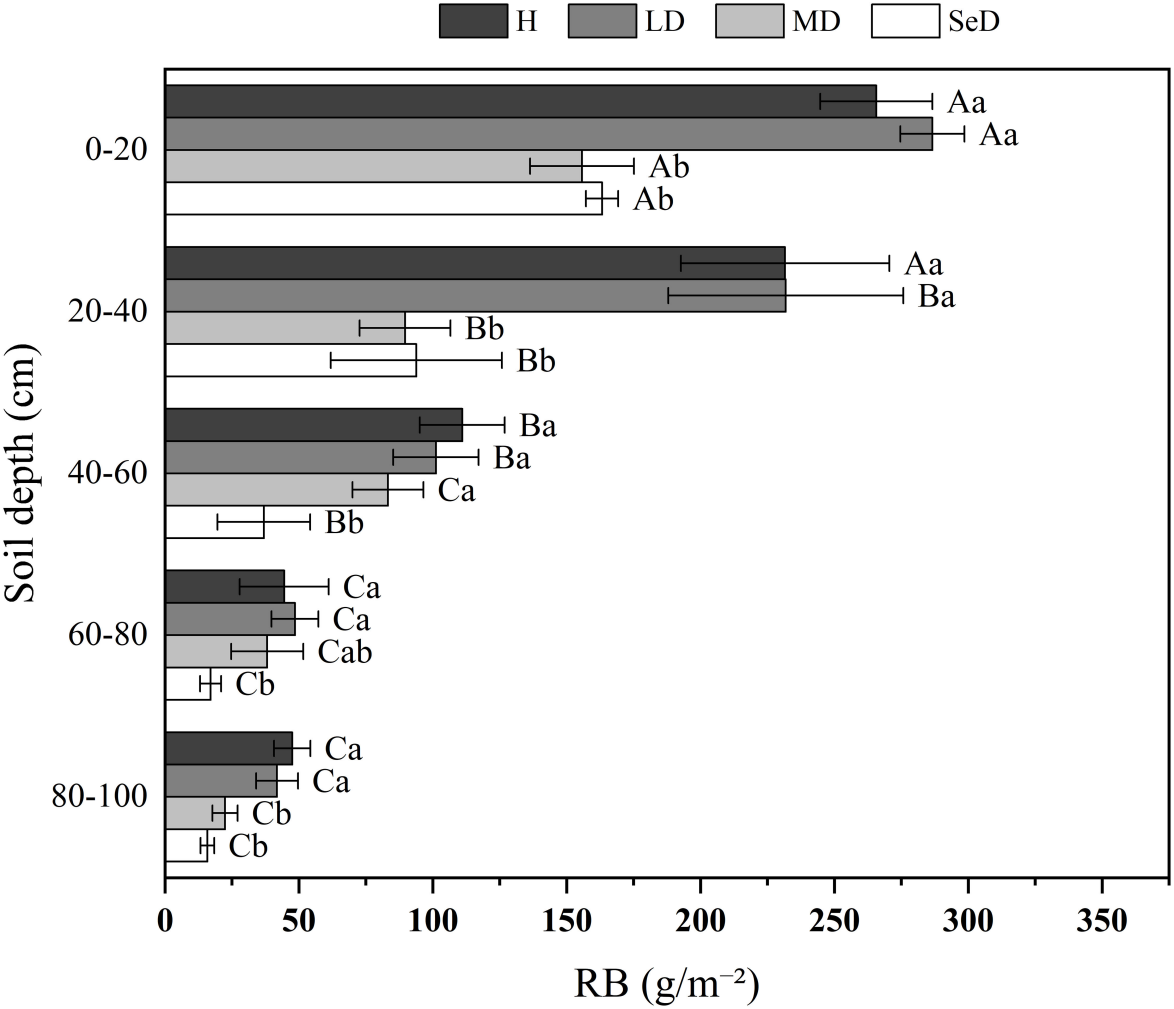
Fine-root biomass characteristics under different degradation levels. Different lowercase letters indicate significant differences among degradation levels within the same soil layer (P < 0.05), whereas the same lowercase letters indicate no significant differences. Different uppercase letters indicate significant differences among soil layers within the same degradation level (P < 0.05), whereas the same uppercase letters indicate no significant differences. RB denotes fine-root biomass. H, LD, MD, and SeD represent healthy, lightly degraded, moderately degraded, and severely degraded plots, respectively.

Fine-root biomass (RB) showed significant variation along the 0–100 cm soil profile across degradation levels and generally decreased as degradation intensified (**Figure 4**). Across all soil layers, fine-root biomass was generally higher in the H and LD plots but significantly lower in the MD and SeD plots, with the greatest differences occurring in the 0–20 cm layer. Vertically, fine-root biomass declined with increasing soil depth across all degradation levels. The 0–40 cm layer constituted the main zone of fine-root distribution, and biomass decreased markedly below 60 cm.

In summary, the Moderately degraded stage is the phase during which changes in fine-root chemical composition and biomass are more pronounced in *Populus simonii*.

### 3.2 Characteristics of soil nutrient changes under poplar shelterbelts at different degradation levels

**Figure 5:**
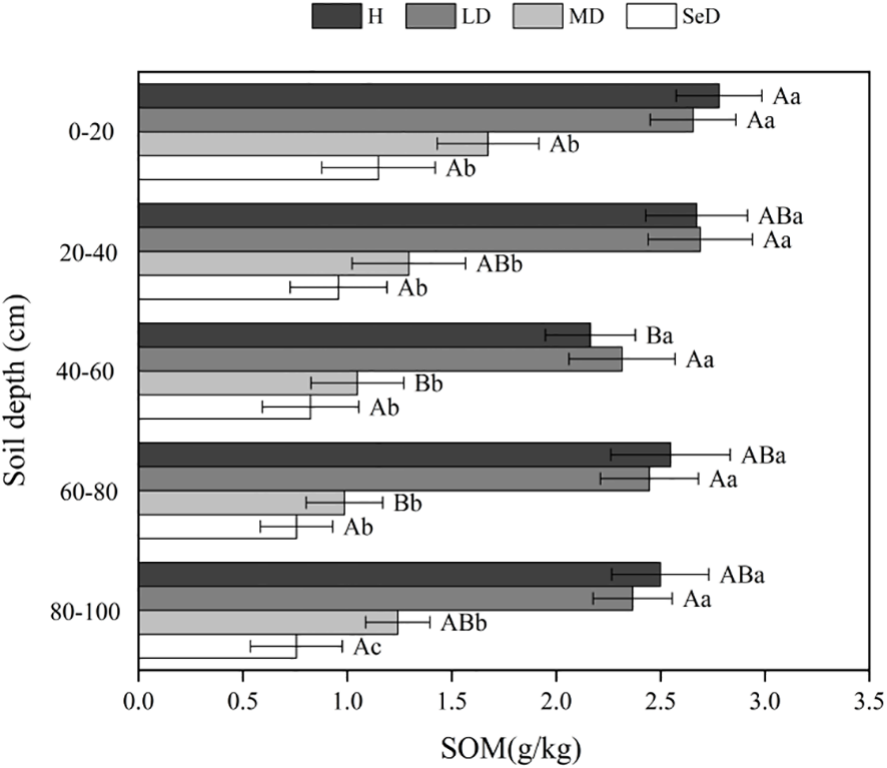
Soil organic matter characteristics at different soil depths under different degradation levels. Different lowercase letters indicate significant differences among degradation levels within the same soil layer (P < 0.05), whereas the same lowercase letters indicate no significant differences. Different uppercase letters indicate significant differences among soil layers within the same degradation level (P < 0.05), whereas the same uppercase letters indicate no significant differences (P > 0.05). H, LD, MD, and SeD represent healthy, lightly degraded, moderately degraded, and severely degraded plots, respectively.

Soil organic matter (SOM) content generally decreased with soil depth, with significant differences observed among degradation levels and soil layers (**Figure 5**). The topsoil (0–20 cm) had the highest SOM content. Across degradation levels, SOM content was significantly higher in the Healthy and Lightly degraded plots than in the Moderately degraded and Severely degraded plots. Total nitrogen (TN) and alkali-hydrolyzable nitrogen (AN) showed distribution patterns similar to that of SOM, decreasing with soil depth and generally being higher in the Healthy and Lightly degraded plots than in the Moderately degraded and Severely degraded plots (**Figures 2a, d**). Total phosphorus (TP) varied relatively little among soil layers, but its content in Healthy plots was generally higher than that in the other degradation levels (**Figure 2e**). Available phosphorus (AP) showed a distribution pattern distinct from those of the other nutrients, with lower values in the surface layer and the most pronounced differences occurring in the deep layer (80–100 cm). Specifically, deep-layer AP content was higher in the Severely degraded plots, whereas the Healthy and Lightly degraded plots maintained lower levels across all soil layers (**Figure 2b**). Total potassium (TK) showed little variation with soil depth, and differences among degradation levels were not significant (**Figure 2f**). Available potassium (AK) was higher in the surface layer (0–40 cm) and decreased with increasing soil depth, and surface-layer AK levels were generally higher in the Healthy and Lightly degraded plots than in the Moderately degraded and Severely degraded plots (**Figure 2c**). In summary, nutrient differentiation is more pronounced during the Moderately degraded stage of *Populus simonii* shelterbelts.

**Figure 6:**
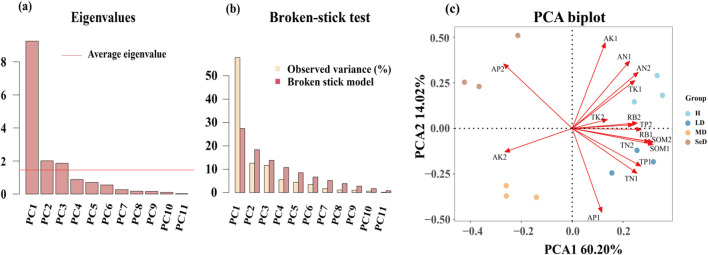
Principal component analysis (PCA) biplot of soil nutrient and root-trait characteristics across degradation levels. **(a)** eigenvalue plot; **(b)** broken-stick test; **(c)** PCA biplot. H, LD, MD, and SeD denote healthy, lightly degraded, moderately degraded, and severely degraded plots, respectively. A variable suffix of “1” indicates the mean value of indicators in the 0–20 cm soil layer, whereas a suffix of “2” indicates the mean value of indicators in the 20–100 cm soil layer. Red arrows represent root-related indicators, and blue arrows represent soil nutrient factors. Arrow direction indicates the direction of variable change, and arrow length reflects the strength of the correlation between the variable and the ordination axes.

The PCA results indicate that the first two principal components effectively captured the differentiation characteristics of plots under different degradation levels. The scatter plot showed that PC1 had the highest eigenvalue, and the broken-stick test indicated that the actual explanatory power of PC1 exceeded random expectation (**Figures 6a, b**). Plots representing different degradation levels showed a clear tendency to separate in the ordination space (**Figure 6c**). Healthy and Lightly degraded plots were primarily distributed along the positive direction of PC1 and were closely associated with indicators such as AK1, AN1, AN2, TK1, TP2, RB1, RB2, SOM1, and SOM2, whereas Moderately degraded and Severely degraded plots were mainly distributed along the negative direction of PC1 and were more closely associated with indicators such as AP2 and AK2. Overall, plot differentiation across degradation levels was primarily driven by the overall nutrient gradient, with PC1 serving as the dominant axis separating the different degradation states.

### 3.3 Relationship between fine-root traits of *Populus simonii* and understory soil nutrients under different degradation levels

**Figure 7:**
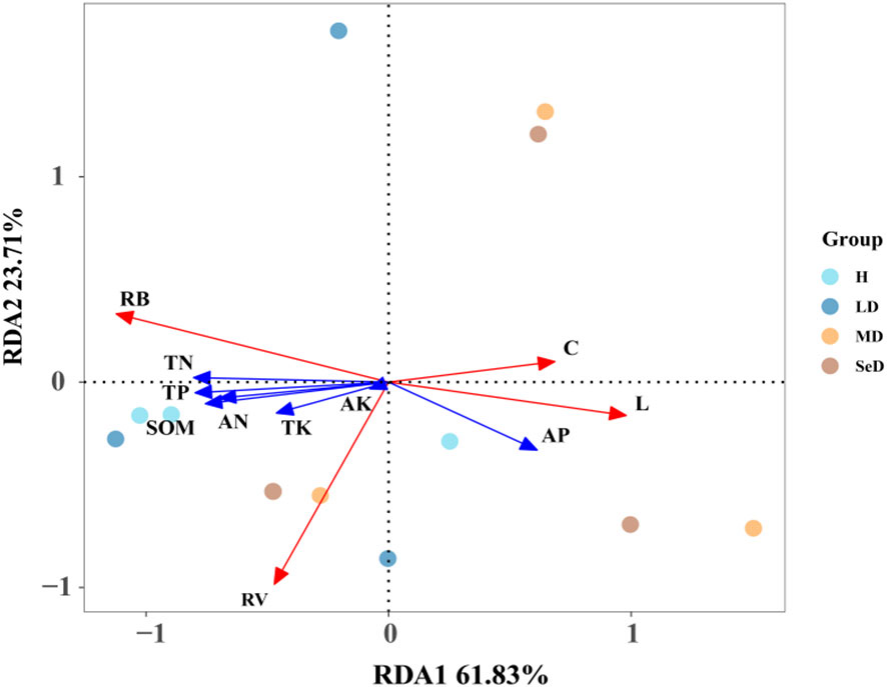
RDA ordination plot of soil nutrient factors and fine-root trait indicators under different degradation levels. Sampling points of different colors represent plots under different degradation levels, where H, LD, MD, and SeD denote healthy, lightly degraded, moderately degraded, and severely degraded plots, respectively. Blue arrows represent soil nutrient factors, and red arrows represent fine-root trait indicators. Arrow direction indicates the direction of variable change in ordination space, whereas arrow length reflects the strength of the correlation between variables and the ordination axes.

The RDA ordination results indicate (**Figure 7**) that plots under different degradation levels show a certain degree of separation in the ordination space. Specifically, Healthy and Lightly degraded plots are mainly distributed in the negative quadrants of RDA1 and are associated with soil organic matter, total nitrogen, total phosphorus, alkali-hydrolyzable nitrogen, and total potassium, whereas Moderately degraded and Severely degraded plots are mainly distributed in the positive quadrants of RDA1 and are associated with lignin, cellulose, and available phosphorus. The soil factor fitting results (**Table 2**) show that total nitrogen, total phosphorus, and alkali-hydrolyzable nitrogen are significantly correlated with the ordination pattern (P < 0.05), whereas soil organic matter shows marginal significance (P = 0.056). These results indicate that total nitrogen, total phosphorus, and alkali-hydrolyzable nitrogen are the key soil variables driving the differentiation of fine-root trait indicators. The fitting results for fine-root trait variables (**Table 3**) indicate that fine-root activity, lignin, cellulose, and biomass are all significantly correlated with the ordination pattern, with fine-root activity showing the strongest fit (r² = 0.9594, P = 0.001). In terms of variable direction, fine-root activity and biomass were generally aligned with the overall soil nutrient gradient, whereas lignin and cellulose were oriented in the opposite direction, indicating a significant correspondence between changes in the soil nutrient background during degradation and variation in fine-root activity, biomass, and structural components.

**Table 2:** Significance test results for soil factor fitting.

Soil factor	RDA1	RDA2	r²	P
SOM	-0.9971	-0.0764	0.4482	0.056
AN	-0.9980	-0.0641	0.5295	0.038^*^
AP	0.9040	-0.4276	0.4160	0.095
AK	-0.9961	-0.0878	0.0064	0.972
TN	-0.9953	0.0973	0.5408	0.025^*^
TP	-0.9995	-0.0311	0.5681	0.021^*^
TK	-0.9730	-0.2308	0.1966	0.358

RDA1 and RDA2 represent the coordinates of soil factors in the redundancy analysis ordination space; r² indicates the strength of the relationship between environmental factors and the ordination pattern; P denotes the significance level obtained from a 999-permutation test. * indicates P < 0.05. Significance values were rounded to three decimal places.

**Table 3:** Results of the significance test for fine-root trait indicators.

Fine-root trait	RDA1	RDA2	r²	P
RV	-0.3797	-0.9251	0.9594	0.001^***^
C	0.9998	-0.0219	0.5073	0.035^*^
L	0.9926	-0.1217	0.7756	0.002^**^
RB	-0.9554	0.2954	0.6993	0.005^**^

RDA1 and RDA2 represent the coordinates of fine-root trait indicators in the ordination space of redundancy analysis; r² indicates the strength of the relationship between environmental factors and the ordination pattern; P denotes the significance level obtained from a 999-permutation test. ^*^, ^**^, and ^***^ denote P < 0.05, P < 0.01, and P < 0.001, respectively. Significance values are reported to three decimal places. RV, fine-root activity; C, cellulose; L, lignin; RB, fine-root biomass.

### 3.4 Mechanisms underlying soil nutrient–root trait interactions during degradation

**Figure 8:**
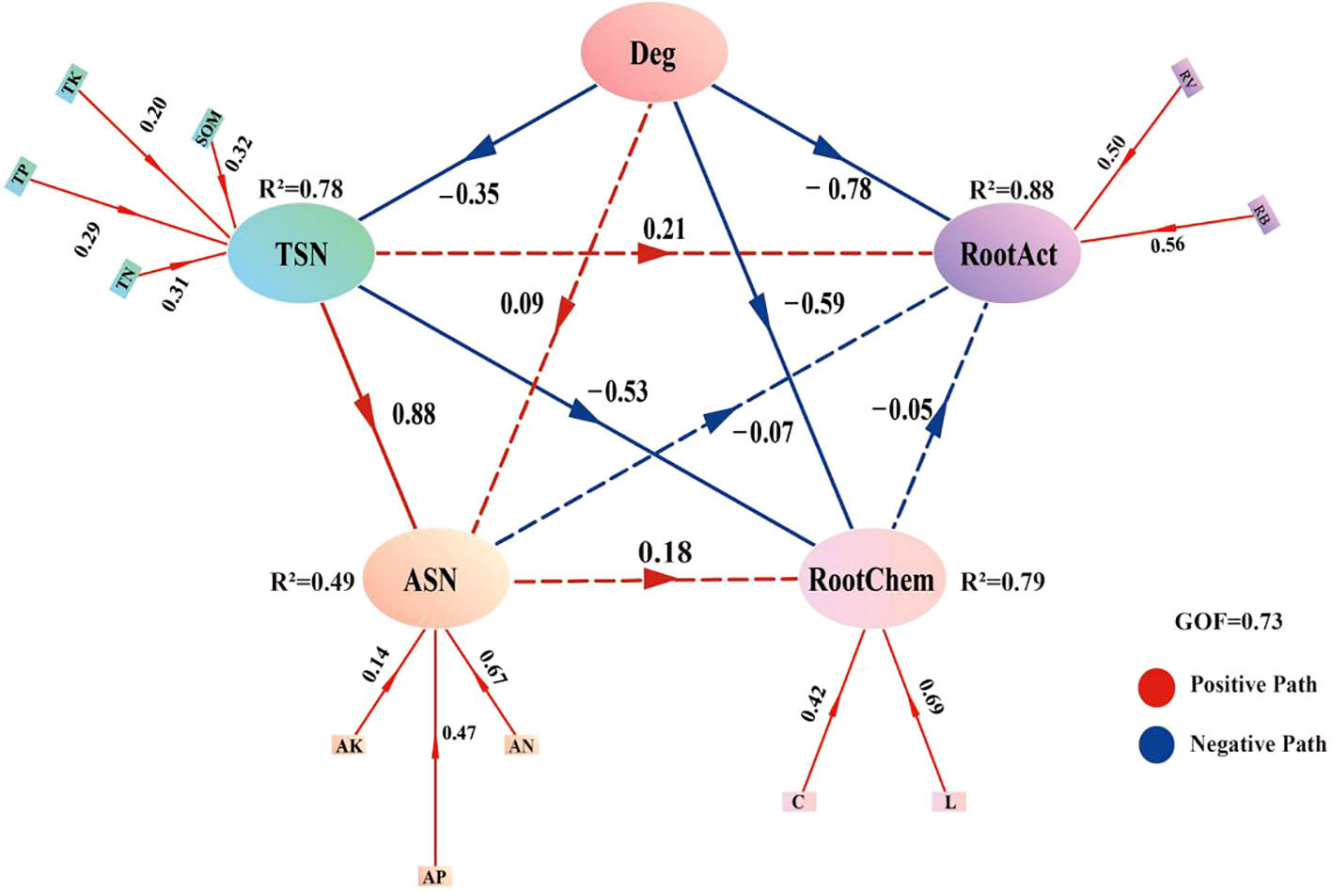
Partial least squares structural equation modeling (PLS-SEM) showing the relationships among degradation level, soil nutrients, and fine-root traits. Deg denotes degradation level; TSN denotes total soil nutrients, including soil organic matter (SOM), total nitrogen (TN), total phosphorus (TP), and total potassium (TK); ASN denotes available soil nutrients, including alkali-hydrolyzable nitrogen (AN), available phosphorus (AP), and available potassium (AK); RootChem denotes fine-root chemical composition, including lignin (L) and cellulose (C); RootAct denotes fine-root functional performance, including fine-root activity and biomass. Red arrows indicate positive pathways, whereas blue arrows indicate negative pathways; solid lines indicate significant pathways, and dashed lines indicate non-significant pathways. Numbers next to the arrows represent standardized path coefficients. The R² values shown next to the ellipses indicate the coefficients of determination for the endogenous latent variables, and GOF indicates the overall goodness of fit of the model.

A path model of “degradation level–soil nutrients–root traits” was constructed using PLS-SEM (**Figure 8**). The model showed acceptable overall performance, with a GoF value of 0.73, exceeding the empirical threshold of 0.70 commonly used to indicate very good performance in PLS-PM (Sánchez, 2013). The path analysis results indicated that degradation level had a significant negative effect on total soil nutrients, suggesting that increasing degradation led to an overall decline in soil organic matter and total nitrogen, phosphorus, and potassium. Degradation level also had a significant negative effect on fine-root activity and fine-root chemical composition, indicating that increasing degradation directly inhibited fine-root activity and drove changes in fine-root chemical composition. Meanwhile, total soil nutrients had a significant positive effect on available nutrients but a significant negative effect on fine-root chemical composition, suggesting that changes in total soil nutrients served as a key mediating pathway linking degradation to fine-root structural responses.

Overall, this path model suggests that increasing degradation was associated with reductions in the total soil nutrient pool, changes in nutrient availability and fine-root chemical composition and reduced fine-root activity. However, because this study was based on observational data, the temporal order and causal direction among stand degradation, soil nutrient depletion, and fine-root trait changes cannot be definitively established.

## 4 Discussion

### 4.1 Fine-root changes in *Populus simonii* across the degradation levels and their ecological significance

Fine roots are important belowground organs through which plants perceive soil stress and regulate resource acquisition, and changes in their activity, biomass, and chemical composition can serve as sensitive indicators of belowground response strategies under adverse conditions (Kalra et al., 2024; Hou et al., 2024). Our results showed that fine-root activity and biomass in *Populus simonii* decreased significantly as degradation intensified, indicating a continuous decline in belowground metabolic activity and resource acquisition capacity. This change was not merely a decrease in a single indicator, but rather indicated that fine-root metabolic activity, biomass accumulation, and structural chemical components changed during degradation, reflecting sustained suppression of belowground functional responses in *Populus simonii*. Such changes may further constrain root growth and expansion and may also affect aboveground growth by weakening water and nutrient acquisition capacity (Freschet et al., 2021; Kou et al., 2022; Lopez et al., 2023).

Meanwhile, this study found that lignin content and the lignin-to-cellulose ratio reached relatively high levels at the Moderately degraded stage, indicating that *Populus simonii* fine roots gradually increased structural investment under resource-limited conditions. Lignin accumulation is considered one of the important mechanisms by which woody plants respond to abiotic stress (Han et al., 2022), as it can enhance cell-wall stability and improve tissue tolerance (Li D, et al., 2022). Recent studies have further shown that the molecular and chemical composition of fine roots is closely related to the root economics spectrum, and that higher lignin and aromatic carbon investment is generally associated with more conservative root resource-use strategies (Wang M, et al., 2024). Thus, the increases in lignin content and the lignin-to-cellulose ratio at the Moderately degraded stage are unlikely to represent isolated chemical changes but rather may reflect a belowground strategic adjustment in *Populus simonii* fine roots from resource acquisition toward maintaining tissue stability.

Notably, fine-root activity and biomass had already declined markedly at the Moderately degraded stage, whereas the proportion of structural components remained relatively high, indicating that fine roots at this stage exhibited a combination of low activity, reduced acquisitive capacity, and high structural investment. Previous studies have shown that fine-root lifespan is closely related to root morphological, physiological, and chemical traits (Eissenstat et al., 2000; McCormack et al., 2015). Under increasing external stress, roots often tend to extend lifespan and reduce turnover. Combined with the results of the present study, this suggests that the Moderately degraded stage may represent an important period during which belowground functioning in *Populus simonii* shifts from adaptive adjustment to marked functional limitation. In other words, fine roots at this stage are characterized not only by reduced quantity, but also by evident changes in their functional role and resource-use strategy.

### 4.2 Soil nutrient differentiation and its role in driving fine-root changes

Soil nutrients provide an important resource basis for maintaining belowground functioning in plantations in sandy areas (Bardgett et al., 2014). Our results showed that soil organic matter, total nitrogen, and alkali-hydrolyzable nitrogen generally declined along the 0–100 cm soil profile during the degradation of *Populus simonii* shelterbelts, with the surface layer showing the strongest response. This pattern is consistent with the common phenomenon in degraded ecosystems of arid regions, where surface soil organic matter and nitrogen tend to decline first. Previous studies have shown that the restoration of artificial vegetation in the Tengger Desert can significantly increase soil organic carbon and total nitrogen stocks, with the most pronounced changes occurring in the surface layer (Li X, et al., 2022). This indirectly suggests that vegetation degradation may first weaken the accumulation of carbon and nitrogen in surface soils. In the Mu Us Sandy Land, soil C, N, and P contents have also been reported to decline markedly with increasing desertification (Ning et al., 2021).

Our results further showed that the effects of degradation were not confined to the surface layer. In the 40–100 cm soil layers, soil organic matter and total nitrogen remained significantly higher in the Healthy and Lightly degraded plots than in the Moderately degraded and Severely degraded plots. Previous studies have shown that mid- and deep-soil layers represent an important component of profile resources, and that changes in soil nutrients cannot be assessed solely on the basis of surface soils (Wang et al., 2016; Smal et al., 2019). Combined with our findings, this suggests that the degradation of *Populus simonii* shelterbelts is manifested not only in declining surface soil nutrient conditions but may also be accompanied by the weakening of soil resource conditions in mid- and deep-soil layers. For artificial shelterbelts such as poplar stands that rely on deeper resources to sustain growth, the weakening of soil resource conditions in mid- and deep-soil layers may further constrain belowground resource acquisition, thereby aggravating limitations on belowground functioning (Imada et al., 2008; Germon et al., 2020).

Soil nutrient differentiation in the study area may arise from two complementary processes. On the one hand, as canopy closure declines and the number of declining individuals increases, litter and root inputs are reduced, thereby weakening soil organic matter formation and nutrient replenishment (Song et al., 2024). On the other hand, reduced vegetation cover may enhance the selective transport of fine particles and organic matter-enriched fractions in the surface soil by wind erosion, thereby accelerating the loss of surface resources (Zhao et al., 2022). The PCA results further integrated these differences into a comprehensive nutrient gradient, indicating that clear differentiation in soil nutrient conditions had developed among plots with different degradation levels. This differentiation was mainly reflected in the pronounced contrast between the Healthy and Lightly degraded plots and the Moderately degraded and Severely degraded plots.

The RDA results indicated that total nitrogen, total phosphorus, and alkali-hydrolyzable nitrogen were the key soil factors influencing fine-root variation. Fine-root activity and biomass were aligned with the overall nutrient gradient, whereas lignin and cellulose showed the opposite trend. This suggests that what matters most during degradation is not the absolute level of any single nutrient, but rather the re-differentiation of soil resources along the profile and its influence on fine-root allocation and resource-use strategies (Jobbágy and Jackson, 2001). As an essential basis for protein synthesis and root metabolism, nitrogen decline can directly restrict fine-root activity and growth. Phosphorus, in contrast, is closely related to energy metabolism and the maintenance of cell structure, and changes in phosphorus availability may affect root investment in structural components (Khan et al., 2023). Previous studies have shown that changes in soil nutrient conditions can significantly affect fine-root biomass in poplar plantations and further influence its distribution along the soil profile (Fortier et al., 2019). Therefore, the soil nutrient differentiation observed in this study likely drives fine roots from a relatively acquisitive state toward a more conservative maintenance state by altering resource supply patterns and the availability of plant-accessible nutrients. Overall, soil nutrient differentiation is not only a consequence of vegetation degradation but may also represent an important environmental context shaping belowground fine-root responses.

### 4.3 A belowground interpretation of stand degradation based on fine-root trait changes and soil nutrient differentiation

Taken together, these results suggest that the degradation of *Populus simonii* shelterbelts is manifested not only in weakened aboveground growth and deteriorated stand structure but is also accompanied by concurrent fine-root changes and soil nutrient differentiation belowground, which may in turn lead to changes in belowground ecosystem functioning (Hartmann et al., 2020; Comas et al., 2013). As degradation intensified, fine-root activity and biomass declined continuously, indicating a progressive weakening of belowground resource acquisition capacity. Meanwhile, soil organic matter, total nitrogen, and alkali-hydrolyzable nitrogen generally declined along the soil profile, suggesting that soil resource conditions beneath the stands were also continuously deteriorating. These two processes showed consistent trends across the degradation levels and were significantly associated in the RDA and PLS-SEM analyses, indicating that fine-root changes and soil nutrient differentiation jointly contribute to the belowground processes underlying stand degradation.

More importantly, these belowground changes are unlikely to represent isolated fluctuations in individual indicators, but more likely reflect an overall adjustment of the resource acquisition system during stand degradation (Bergmann et al., 2017). On the one hand, declines in fine-root activity and biomass imply a reduced capacity of plants to acquire water and nutrients belowground. On the other hand, the weakening of soil resource conditions further constrains the resource space available to fine roots (Fort et al., 2017). Particularly at the Moderately degraded stage, structural investment in fine roots increased whereas metabolic activity declined markedly, and soil nutrient differentiation also became more pronounced, suggesting that this stage may represent an important turning point in belowground functioning. In other words, the Moderately degraded stage is not merely an intermediate state within the degradation sequence but may instead constitute a critical phase during which belowground resource-use strategies shift from relatively acquisitive to more conservative maintenance strategies.

Therefore, from a belowground perspective, the degradation of *Populus simonii* shelterbelts can be understood as a process in which reduced fine-root resource acquisition capacity and deteriorating soil resource conditions progress simultaneously. The former lowers the plant’s capacity to utilize belowground resources, whereas the latter further constrains resource supply conditions. Together, these two processes constitute an important belowground basis for stand degradation. Accordingly, the diagnosis of degradation in artificial shelterbelts in sandy areas should not rely solely on aboveground tree performance, but should also incorporate belowground indicators, including fine-root activity, biomass, and soil nutrient variation along the profile.

## 5 Conclusion

This study focused on *Populus simonii* shelterbelts across different degradation levels in the Kubuqi Desert, systematically analyzed changes in fine-root traits and the decline characteristics of understory soil nutrients, and revealed the coordinated response patterns of belowground systems during the degradation process. This study showed that the degradation of *Populus simonii* shelterbelts was accompanied by continuous declines in fine-root activity and biomass, as well as increases in lignin content and the lignin-to-cellulose ratio, indicating a shift in fine-root resource-use strategy from a relatively acquisitive state to a more conservative maintenance state. Meanwhile, soil organic matter, total nitrogen, and alkali-hydrolyzable nitrogen generally declined along the 0–100 cm soil profile, with the surface layer showing the strongest response, indicating that degradation effects had gradually extended from the surface to the mid- and deep-soil layers. Overall, fine-root trait changes were significantly associated with soil nutrient profile differentiation, and the Moderately degraded stage exhibited a more pronounced transition in belowground functioning. These findings provide a basis for identifying belowground processes and supporting the ecological restoration of degraded shelterbelts in sandy areas.

## Data Availability

The raw data supporting the conclusions of this article will be made available by the authors, without undue reservation.
