# Supplementary material for: Associations between fine-root traits and soil nutrient profile differentiation during degradation of Populus simonii shelterbelts in sandy areas
Source: Front Plant Sci. 2026 Jul 9;17:1865203. doi: 10.3389/fpls.2026.1865203 (PMC13391941; doi:10.3389/fpls.2026.1865203)
Supplement: Supplementary file 1 [file Table1.docx]

Supplementary Table S1. Soil nutrient contents across degradation levels and soil depths based on one-way ANOVA followed by Tukey’s multiple comparisons

| **Indicator** | **Soil depth (cm)** | **H** | **LD** | **MD** | **SeD** |
| --- | --- | --- | --- | --- | --- |
| SOM  g/kg | 0-20 | 2.78 ± 0.20Aa | 2.66 ± 0.21Aa | 1.67 ± 0.24Ab | 1.15 ± 0.27Ab |
|  | 20-40 | 2.67 ± 0.24ABa | 2.69 ± 0.25Aa | 1.29 ± 0.27ABb | 0.96 ± 0.23Ab |
|  | 40-60 | 2.16 ± 0.22Ba | 2.31 ± 0.25Aa | 1.05 ± 0.22Bb | 0.82 ± 0.23Ab |
|  | 60-80 | 2.55 ± 0.29ABa | 2.45 ± 0.23Aa | 0.99 ± 0.18Bb | 0.76 ± 0.17Ab |
|  | 80-100 | 2.50 ± 0.23ABa | 2.37 ± 0.19Aa | 1.24 ± 0.15ABb | 0.76 ± 0.22Ac |
| AN  mg/kg | 0-20 | 23.06 ± 2.02BCa | 23.01 ± 0.62BCa | 17.78 ± 0.75Ac | 20.03 ± 0.49Ab |
|  | 20-40 | 26.18 ± 1.11Aa | 26.47 ± 0.76Aa | 17.82 ± 0.49Ac | 20.43 ± 0.20Ab |
|  | 40-60 | 21.74 ± 0.82ABab | 23.25 ± 0.32Ba | 16.94 ± 0.45Ac | 18.90 ± 0.32Bb |
|  | 60-80 | 24.89 ± 0.88Aa | 21.61 ± 0.88BCb | 17.54 ± 0.42Ac | 19.16 ± 0.14BCc |
|  | 80-100 | 22.40 ± 1.05BCa | 21.50 ± 0.90Cab | 17.79 ± 0.41Abc | 18.41 ± 0.28Cc |
| AP  mg/kg | 0-20 | 1.17 ± 0.08Ba | 1.12 ± 0.10Aa | 1.26 ± 0.11Ca | 0.83 ± 0.03Db |
|  | 20-40 | 1.33 ± 0.05Bd | 1.14 ± 0.06Ac | 2.19 ± 0.06Ba | 1.92 ± 0.04Cb |
|  | 40-60 | 1.21 ± 0.11Bb | 0.56 ± 0.10Bc | 2.08 ± 0.14Ba | 2.18 ± 0.11Ba |
|  | 60-80 | 1.16 ± 0.07Cb | 0.42 ± 0.04Bc | 2.17 ± 0.17Ba | 2.33 ± 0.05Ba |
|  | 80-100 | 2.27 ± 0.10Ab | 1.53 ± 0.45Ac | 2.70 ± 0.12Ab | 3.88 ± 0.18Aa |
| AK  mg/kg | 0-20 | 159.04 ± 8.90Aa | 115.90 ± 3.94Ac | 115.02 ± 4.92Ac | 131.22 ± 7.06Ab |
|  | 20-40 | 155.58 ± 5.55Aa | 127.14 ± 3.68Bb | 91.85 ± 3.77Bc | 92.34 ± 3.64Bc |
|  | 40-60 | 88.17 ± 5.52Ba | 82.75 ± 1.92Ca | 83.27 ± 3.76Ba | 68.30 ± 5.31Bb |
|  | 60-80 | 70.03 ± 5.68Cb | 73.41 ± 2.23Db | 85.88 ± 4.83Ba | 62.62 ± 6.19Bb |
|  | 80-100 | 60.80 ± 5.29Cb | 66.31 ± 0.84Ea | 85.44 ± 5.11Ba | 83.10 ± 8.05Ba |
| TN  g/kg | 0-20 | 0.47 ± 0.03Ab | 0.35 ± 0.05Aa | 0.31 ± 0.05Abc | 0.24 ± 0.02Ac |
|  | 20-40 | 0.45 ± 0.02Aa | 0.36 ± 0.07Aa | 0.25 ± 0.02ABb | 0.21 ± 0.03ABb |
|  | 40-60 | 0.41 ± 0.04Ab | 0.34 ± 0.01Aa | 0.23 ± 0.02Bc | 0.16 ± 0.02ABd |
|  | 60-80 | 0.39 ± 0.02Aa | 0.36 ± 0.03Aa | 0.21 ± 0.01Bb | 0.18 ± 0.02Bb |
|  | 80-100 | 0.39 ± 0.03Aa | 0.36 ± 0.03Aa | 0.23 ± 0.03Bb | 0.21 ± 0.03Bb |
| TP  g/kg | 0-20 | 0.30 ± 0.01Ba | 0.30 ± 0.01Aa | 0.26 ± 0.01Ab | 0.23 ± 0.02Ac |
|  | 20-40 | 0.37 ± 0.02Aa | 0.31 ± 0.01Ab | 0.28 ± 0.01Abc | 0.25 ± 0.01Ac |
|  | 40-60 | 0.37 ± 0.03Aa | 0.30 ± 0.02Ab | 0.26 ± 0.01Ab | 0.26 ± 0.01Ab |
|  | 60-80 | 0.36 ± 0.04Aa | 0.30 ± 0.02Aab | 0.27 ± 0.02Ab | 0.27 ± 0.02Ab |
|  | 80-100 | 0.33 ± 0.01ABa | 0.30 ± 0.01Aab | 0.29 ± 0.03Aab | 0.26 ± 0.03Ab |
| TK  g/kg | 0-20 | 20.80 ± 0.25Aa | 20.58 ± 0.63Aa | 19.49 ± 0.42Aab | 19.97 ± 0.31Ab |
|  | 20-40 | 21.22 ± 0.43Aa | 20.32 ± 0.37Aab | 20.24 ± 0.88Aab | 19.66 ± 0.40ABb |
|  | 40-60 | 20.73 ± 0.47Aa | 20.62 ± 0.21Aa | 20.40 ± 0.21Aa | 19.57 ± 0.19ABb |
|  | 60-80 | 21.11 ± 0.79Aa | 19.95 ± 0.41Aab | 19.20 ± 1.17Ab | 19.03 ± 0.40ABb |
|  | 80-100 | 20.95 ± 0.61Aa | 19.68 ± 0.21Aab | 20.13 ± 0.38Ab | 19.55 ± 0.28Bb |

Note: Data are presented as means ± SE. Different lowercase letters indicate significant differences among degradation levels within the same soil layer, based on one-way ANOVA followed by Tukey’s multiple comparisons (P < 0.05). Different uppercase letters indicate significant differences among soil layers within the same degradation level, based on one-way ANOVA followed by Tukey’s multiple comparisons (P < 0.05). H, LD, MD, and SeD indicate Healthy, Lightly degraded, Moderately degraded, and Severely degraded plots, respectively.
